# Reviewed article: Siqueira PFS, Sousa DBP, Moreira AFG, Marchi BS, Teixeira GC, Martins PF, Alves WA. Deaths from infectious respiratory diseases in the Indigenous population: spatial and time series analysis, Minas Gerais, 2000-2023. Epidemiol Serv Saude. 2026:35;e20250565.

**DOI:** 10.1590/S2237-96222026v35e20250565.c

**Published:** 2026-04-20

**Authors:** Nathalia Sernizon Guimarães

**Affiliations:** 1 Universidade Estadual de Maringá, Departamento de Enfermagem, Maringá, PR, Brazil Universidade Estadual de Maringá Departamento de Enfermagem Maringá PR Brazil

## First round comments 

Prezados autores, 

Agradecemos a submissão do manuscrito intitulado “Tendência temporal e distribuição espacial da mortalidade por doenças respiratórias infecciosas na população indígena em Minas Gerais: análise de séries temporais, 2000-2023”. O estudo aborda tema relevante e pouco explorado, especialmente pela interface entre saúde indígena, epidemiologia e análises espaço-temporais. Contudo, após avaliação detalhada, verificamos que são necessárias modificações substanciais em todas as seções para que o trabalho alcance maior clareza, rigor metodológico e alinhamento com as boas práticas de relato científico. 

Os principais pontos que justificam a decisão de major review incluem: 

Título: 

- Considerando o comentário nos métodos de que o estudo não analisa efetivamente a mortalidade, que implica, por definição, o uso de uma taxa, sugiro readequar o título para: “Tendência temporal e distribuição espacial dos óbitos por doenças respiratórias infecciosas na população indígena em Minas Gerais: análise espacial e de séries temporais, 2000-2023”. 

Resumo: 

A forma como os valores de significância foram inseridos carece de articulação com a narrativa. Recomenda-se reformular trechos como: “Para a análise de tendência, utilizou-se o modelo de regressão por pontos de inflexão (Joinpoint regression)”, e o mesmo se aplica à menção da estatística de Moran, que deve ser devidamente contextualizada. 

Além disso, os resultados estão descritos de maneira abrupta, com a apresentação direta das características predominantes da população estudada, sem uma introdução que situe o leitor. Recomenda-se o uso de conectores e frases de transição que favoreçam a fluidez textual. No que se refere à análise de tendência, sugere-se uma exposição mais objetiva dos achados, indicando claramente as regiões com tendência crescente ou decrescente de mortalidade. 

A expressão “padrão espacial significativo” deve ser melhor explicitada. É essencial indicar onde ocorreram os agrupamentos espaciais (clusters) com maior densidade de óbitos, oferecendo uma descrição geográfica precisa desses padrões. 

Por fim, a frase “alta mortalidade em idosos, desigualdades temporais e territoriais” apresenta-se truncada. Recomenda-se a reescrita para maior completude e coesão, por exemplo: “Os resultados evidenciaram alta mortalidade entre idosos, com expressivas desigualdades temporais e territoriais na distribuição dos óbitos”. 

Os descritores apresentados são suficientes e refletem adequadamente o conteúdo do estudo. 

1- Resumo (métodos): remover a abreviação "(CID-10)". Não é necessário, pois consta uma vez no resumo. Mantenha as abreviações VPA e VPAM somente se constar mais de uma vez no resumo. 

2- Resumo (resultados): inserir dados numéricos (resultado das medidas calculadas) para apoiar as afirmativas. Sugere-se inserir os principais resultados de VPA e VPMA. 

3- Resumo (conclusão): deter-se ao que se pode afirmar a partir dos resultados apresentados no resumo em resposta ao objetivo. 

Introdução: 

- No início da seção, quando os autores contextualizam a problemática das infecções respiratórias, seria oportuno trazer um conceito geral que caracterize esse grupo de doenças, seja pela manifestação clínica, seja pela forma de transmissão. Isso ajudaria o leitor a ter uma familiaridade maior com o entendimento dos autores no que tange às doenças. 

- Embora os autores citem o Fórum Internacional de Sociedades Respiratórias, faltam dados quantitativos globais e nacionais sobre a carga dessas doenças (mortalidade, internações, etc.), como forma de demonstrar a magnitude do problema. Sugiro incluir dados atualizados da Organização Mundial da Saúde (OMS) ou do Global Burden of Disease Study para fortalecer. 

- Os autores mencionam a relação entre a população indígena e as desigualdades em saúde, mas faltam referências mais explícitas sobre as barreiras estruturais enfrentadas, como: acesso geográfico, precariedade de unidades básicas de saúde em áreas indígenas, carência de profissionais e medicamentos, desassistência em vigilância e diagnóstico, etc. 

- No momento em que os autores falam a respeito da aglomeração intradomiciliar, trazendo à tona a quantidade de moradores/habitantes por residência, seria interessante apresentar os dados nacionais e estaduais de densidade domiciliar para o contexto da população geral, como forma de comparar e dar ênfase no valor da população originária. 

- Quando os autores abordam o cenário da covid-19 e da síndrome respiratória aguda grave (SRAG), seria oportuno trazer dados epidemiológicos do cenário da infecção na população geral e indígena, no país e no estado. Essa complementação ajudaria no argumento que os autores tentaram introduzir, no que se referente ao acometimento mais intenso. 

- A justificativa menciona a “escassez de investigações”, mas não explicita o que diferencia este estudo dos demais nem qual a principal lacuna que ele se propõe a preencher. Uma busca em sites de busca retorna vários artigos (https://doi.org/10.1590/1413-812320242912.09392024; https://doi.org/10.1590/S0104-12902022210368pt; https://doi.org/10.1590/S2237-96222025v34e20240176.pt, etc.), então seria importante delimitar o seu diferencial. 

- A introdução transita entre dados demográficos, pandemia, situação sanitária e a estrutura do Sistema Único de Saúde (SUS), mas essas ideias ainda estão pouco articuladas. Nesse sentido, sugiro incluir frases de conexão ou síntese ao final dos parágrafos para orientar o leitor na progressão lógica da argumentação em defesa pelos autores. Recomenda-se a inserção de dados epidemiológicos atualizados que descrevam a incidência e a mortalidade por doenças respiratórias infecciosas entre os povos indígenas, de modo a contextualizar a magnitude do problema nessa população e justificar a relevância do estudo. Ademais, a afirmação de que “a limitada produção sobre o tema reforça a necessidade de evidências que subsidiem ações de saúde mais eficazes e culturalmente adequadas” deve ser respaldada por uma revisão da literatura científica. É importante esclarecer o panorama atual das pesquisas sobre o tema, apontando lacunas do conhecimento, limitações metodológicas de estudos prévios ou a escassez de investigações centradas em contextos socioculturais indígenas. Esse reforço teórico contribuirá para demonstrar a originalidade e a pertinência da proposta. 

Métodos: 

- Os autores mencionam que realizaram um “estudo de análise de série temporal”. Sugiro que seja reformulado para “estudo de séries temporais e múltiplos grupos”, visto que foram realizadas análises em função do tempo e do espaço. Segue uma sugestão leitura a respeito das tipologias de estudo ecológico: https://doi.org/10.1146/annurev.pu.16.050195.000425. 

- Sugiro revisar o uso do termo “mortalidade” ao longo do manuscrito. Considerando que não foram utilizadas estimativas populacionais da população indígena sob risco, o termo pode ser tecnicamente inadequado. Expressões como “óbitos registrados” e “número de óbitos” refletem de forma mais precisa a natureza descritiva dos dados analisados, evitando interpretações equivocadas sobre o risco de morte. 

- Sugiro que os autores revisem as macrorregiões de saúde mencionadas no subitem “local do estudo”. Na minha contagem, foram apresentadas quinze regiões, e não dezesseis, conforme o informado. Analisando a Deliberação CIB/MG n.º 4.394/2023, parece que a região faltante foi a Centro Sul (sugiro revisar o link de acesso na referência pois não funcionou). 

- O estudo considerou apenas a causa básica de morte, conforme codificada na declaração de óbito (DO). No entanto, é possível que doenças respiratórias infecciosas tenham sido registradas como causas associadas ou contribuintes, especialmente em casos com múltiplas comorbidades ou em que a causa básica foi registrada como “insuficiência respiratória”, “sepse”, ou outra complicação genérica. Sugiro pontuar como uma limitação. 

- De acordo com a iniciativa Strengthening the Reporting of Observational Studies in Epidemiology (STROBE), para cada variável do estudo, deve ser fornecida as categorias de respostas. Por exemplo, ao apresentar as variáveis de caracterização (sexo, faixa etária, estado civil, etc.), seria importante complementar: sexo (masculino; feminino), etc. 

- Embora os autores indiquem que os critérios de inclusão visaram reduzir viés, é importante observar que a exclusão de registros com causas associadas, etiologia inconclusiva ou raça/cor ignorada pode introduzir viés de seleção e de informação, especialmente em populações com menor acesso ao diagnóstico ou ao adequado preenchimento da DO. Recomendo reavaliar esse trecho e reconhecer essa limitação de forma mais explícita na discussão. 

- É importante refletir sobre a suposição de homoscedasticidade. Trata-se de uma série temporal de óbitos, na qual é comum haver autocorrelação serial entre os anos consecutivos. A ausência de uma verificação explícita da presença de autocorrelação serial nos resíduos pode comprometer a validade dos intervalos de confiança e dos testes de significância, superestimando os erros tipo I (https://doi.org/10.5123/S1679-49742015000300024). 

- A escolha de utilizar o número absoluto de óbitos como variável dependente na regressão joinpoint, embora operacionalmente mais simples, pode comprometer a interpretação dos resultados, uma vez que não considera variações no tamanho da população indígena ao longo dos anos e entre regiões. Recomendo que os autores justifiquem essa escolha metodológica, especialmente considerando que a literatura recomenda o uso de taxas de mortalidade. 

- Recomenda-se explicitar o critério estatístico usado para a escolha do modelo na regressão joinpoint (como critério de informação bayesiano ponderado ou teste de permutação) na definição do número de segmentos (joinpoints) em cada série histórica analisada, a fim de garantir transparência e reprodutibilidade da análise. 

- A imputação de valores artificiais (0,10) nos anos com ausência de óbitos pode ser metodologicamente questionável. Tal abordagem pode distorcer as estimativas de tendência e amplificar as variações em séries com baixa frequência de eventos. Sugiro que os autores considerem, como alternativa, o uso de média móvel de três pontos, técnica comumente adotada para suavizar flutuações aleatórias em séries de dados raros. 

- Recomendo disponibilizar como material suplementar a lista de códigos da Classificação Internacional de Doenças (10ª edição) utilizados para classificar o agente etiológico e o sítio anatômico da infecção, com indicação de como cada código foi alocado. Isso ajudaria na transparência da categorização adotada, facilitando o entendimento. 

- A divisão espacial por três grandes períodos (2000-2009, 2010-2019, 2020-2023) carece de justificativa teórica ou empírica. Por que esses cortes e não outro (ex.: considerando inflexões reais observadas na série)? Nesse sentido, como as análises já foram realizadas, sugiro justificar os critérios usados para definir os períodos da análise espacial (três blocos). 

- Embora o uso das macrorregiões de saúde seja coerente com o planejamento estadual, seria interessante discutir a limitação do uso dessas divisões administrativas para representar a distribuição real da população indígena, que se organiza territorialmente a partir de terras indígenas e distritos sanitários especiais indígenas. Seria interessante sinalizar nos mapas as regiões em que há esses agrupamentos, por meio de algum centroide. 

- Os autores mencionam o uso de “teste de proporções” nas análises de comparação das características. Recomendo especificar o nome exato do teste estatístico utilizado (ex.: teste Z, teste qui-quadrado de Pearson ou teste exato de Fisher, conforme os pressupostos), a fim de garantir clareza metodológica e reprodutibilidade do estudo. 

- utilizar para qualificação do relatório de pesquisa o RECORD (Reporting of studies Conducted using Observational Routinely-collected health Data), que é uma extensão do guideline STROBE, voltada especificamente para o relato de estudos observacionais que utilizam dados de saúde rotineiramente coletados. Isso garantirá maior coesão e transparência à pesquisa. 

Especificar que se trata de um estudo ecológico de agregados temporais e especiais. Ainda no delineamento do estudo, já delimitar qual a unidade de análise foi utilizada na pesquisa. 

Na fonte de dados, informar se foi utilizado o Tabnet ou Tabwin para extração de dados e especificar se já foram qualificados ou ainda existem informações preliminares. Dentre os critérios de seleção, incluíram-se aqueles que tiveram como causa de morte “não especificadas”. Nesse caso, os autores podem afirmar que esses casos são referentes às doenças respiratórias infecciosas? 

Na análise de dados, a substituição da ausência de óbitos por valores artificiais (como 0,10) nas séries temporais deve ser tecnicamente justificada, uma vez que essa prática pode introduzir viés nas estimativas e comprometer a validade dos resultados. Embora o software Joinpoint aceite valores zero, o uso de log-transformação (comum nesse tipo de análise) torna o log(0) indefinido, razão pela qual alguns optam por inserir valores próximos de zero apenas nesse contexto específico. No entanto, tal decisão metodológica precisa ser explicitada de forma clara no manuscrito, preferencialmente acompanhada de referência que fundamente a escolha. Recomenda-se, ainda, que os sejam discutidos os possíveis impactos dessa imputação artificial nos resultados e nas interpretações da tendência temporal. 

Ainda, sugere-se que os autores apresentem a justificativa para a agregação dos anos utilizada na análise espacial, explicitando os critérios que fundamentaram a divisão em três estratos temporais (2000-2009, 2010-2019 e 2020-2023). É importante esclarecer se essa cisão baseou-se em aspectos epidemiológicos, operacionais ou na distribuição dos dados ao longo do tempo. Ademais, não está claro se a distribuição espacial foi realizada com base na média de óbitos por município (com objetivo de suavizar variações) ou se foram considerados os números absolutos de óbitos em cada período. Essa informação é essencial, sobretudo para a correta interpretação de padrões espaciais e indicadores derivados, como taxas padronizadas ou medidas de autocorrelação espacial. Em relação ao método estatístico, solicita-se que os autores especifiquem o número máximo de pontos de inflexão (joinpoints) testados no modelo, uma vez que essa decisão impacta diretamente a complexidade da regressão segmentada e a sensibilidade na detecção de mudanças na tendência. Ademais, não está claro se os dados inseridos no modelo correspondem a valores absolutos de óbitos, médias anuais ou taxas padronizadas por população - informação essencial para a adequada interpretação dos resultados. Embora o manuscrito mencione que slopes com p-valor superior ao nível de significância podem ser considerados para fins de segmentação, não foi explicitado qual valor-p foi adotado como critério para inferência estatística. Por fim, caso tenham sido calculadas taxas ou coeficientes de mortalidade, é imprescindível indicar qual base populacional foi utilizada como denominador, bem como a fonte dessas estimativas (por exemplo, IBGE ou DATASUS). 

Resultados: 

- Embora a seção de resultados esteja bem detalhada e apresente adequadamente os dados obtidos, observa-se que há um excesso de dados numéricos descritos de forma textual, o que torna a leitura por vezes densa e exaustiva. Considerando que os valores absolutos e percentuais já estão organizados de maneira clara nas tabelas, recomenda-se que a descrição no texto seja mais sintética, destacando apenas os achados mais relevantes e significativos. 

- O modelo joinpoint gerou resultados detalhados, mas falta uma síntese geral das tendências predominantes e regiões com padrões divergentes. Sugiro acrescentar uma frase-resumo para referenciar a tabela com as tendências, destacando quais regiões apresentaram aumento, redução ou estabilidade - e em que períodos, com foco nos padrões e nas divergências. 

- Caso seja mantida, a imputação de 0,10 óbito em anos sem registros, embora metodologicamente citada, deve ser interpretada com cautela nos resultados, para que o leitor não os entenda como eventos reais. Recomendo sinalizar de forma clara como nota de rodapé da tabela quais foram as séries históricas que precisaram ter valores imputados. 

- Nas tabelas em que são empregados os testes de hipótese para comparação das frequências, sugiro que os autores indiquem/sinalizem, por meio de símbolo sobrescrito/letra sobrescrita qual foi o teste aplicado em cada situação, a fim de garantir clareza metodológica e reprodutibilidade do estudo (similar ao feito na Tabela 2). 

- A Tabela 3 está um pouco confusa. A coluna “classificação da tendência”, ao se referir à variação anual, deveria estar imediatamente após ela. Uma opção seria colocar apenas como nota: “Valores positivos de variação percentual, desde que estatisticamente diferentes de zero (considerando o intervalo de confiança), indicam tendência crescente; valores negativos indicam tendência decrescente. Os demais foram interpretados como tendência estacionária”. 

- Recomendo rever a apresentação conjunta de p-valores e intervalos de confiança nas estimativas de tendência, uma vez que ambos expressam a significância estatística da mesma forma. Quando o intervalo de confiança é apresentado (preferível, inclusive), o p-valor pode ser dispensado, evitando redundância na exposição dos resultados e contribuindo para uma apresentação mais concisa, interpretativa (magnitude e significância) e objetiva. 

- Nas Figuras 1 e 2, além da sugestão de inserção dos centroides indicativos das terras indígenas ou dos distritos sanitários especiais indígenas, recomenda-se a sobreposição de uma nova camada geográfica delimitando as dezesseis macrorregiões de saúde. Essa demarcação contribuiria para alinhar visualmente os dados espaciais às análises temporais, que foram estratificadas por macrorregião, favorecendo a coerência entre os diferentes eixos analíticos. 

Tabela 1: sugere-se inserir as categorias abaixo das variáveis. 

Tabela 1: remover os termos repetidos (anos) das categorias da variável "Faixa etária (anos)". 

Tabela 2: células com valor igual a zero devem ser indicadas apenas por "-" 

Tabela 2: todos os dados das células devem ter a mesma natureza do que foi informado no cabeçalho da tabela. Não é permitido, por exemplo, em uma tabela cujo cabeçalho informa contagens, incluir p-valor. Sugere-se remover a linha p-valor. 

Tabela 3: ajustar as células da coluna VPA que apresentam "- (-)". Inserir somente "-" ao invés de "- (-)" 

Figura 1, 2: ajustar o título e identificar cada parte, conforme instrução. Para figuras compostas (mosaicos), identifique cada parte com letras maiúsculas e descreva-as na legenda, em texto completo de sentido (ex.: “Consumo de medicamentos em homens (A) e mulheres (B)”). Preferencialmente empregue a mesma escala nas figuras que compõem o mosaico. Minimize o número de elementos dentro do campo de dados e certifique-se de que todos estejam claramente identificados. 

Figuras 1, 2: se possível, aumentar um pouco o tamanhos dos mapas e a resolução. Algumas partes apresentam difícil visualização. 

Discussão: 

- Recomendo que a seção de discussão seja iniciada com um parágrafo-síntese dos principais achados do estudo, conforme as orientações da iniciativa STROBE. Esse parágrafo deve retomar de forma concisa os resultados mais relevantes da análise temporal e espacial da mortalidade, sem repeti-los em detalhes/valores, servindo como base para a interpretação e a discussão aprofundada nos parágrafos subsequentes. 

- O texto menciona referências, mas não explora contrastes ou convergências relevantes com a literatura. Sugiro discutir se os padrões identificados (ex.: maior mortalidade na região “x”, predominância de causas não especificadas, etc.) são similares ou divergentes dos encontrados em outros estados, em populações indígenas não mineiras, ou na população geral. 

- Algumas macrorregiões apresentaram tendências estacionárias ou oscilações nos indicadores sem um padrão claro ou consistente ao longo do tempo. Recomendo que os autores explorem possíveis explicações para esses comportamentos, como a ocorrência de subnotificações ou falhas no preenchimento da DO, a eventual migração de indivíduos indígenas entre municípios ou estados, ou ainda diferenças na cobertura e acesso aos serviços de saúde. 

- A discussão toca em vulnerabilidades sociais e em estratégias de proteção social, mas de modo superficial. Sugiro explorar mais o papel da precariedade estrutural (saneamento, água, barreiras geográficas e linguísticas, preconceito, etc.) e como isso pode se traduzir em maior mortalidade, bem como discutir as estratégias governamentais para atenuar esses contextos. 

- O texto menciona a necessidade de políticas públicas, mas fica um pouco vago. Pode-se indicar o que os achados implicam para a vigilância epidemiológica, para a atuação da Secretaria Especial de Saúde Indígena, para o fortalecimento da atenção primária nas áreas indígenas, ou mesmo para qualificação da DO. 

- A discussão aborda limitações (ex.: dados secundários), mas poderia ser mais autocrítica. Recomendo apontar que a análise se restringiu à causa básica, não utilizou taxas padronizadas por falta de dados populacionais indígenas por município e que a heterogeneidade dos dados pode afetar a robustez das tendências, além de outras reflexões pontuadas nos métodos. 

- Dado o recorte étnico-racial adotado, sugiro que a discussão inclua uma reflexão sobre a importância da qualificação dos sistemas de informação para a população indígena, especialmente no que se refere ao preenchimento da variável raça/cor e à inclusão de marcadores territoriais mais sensíveis à organização sociocultural indígena. 

- Recomendo finalizar a discussão indicando que os achados podem subsidiar estudos futuros mais aprofundados sobre mortalidade indígena, de forma mais enfática e propositiva, bem como ações específicas de vigilância e planejamento em saúde voltadas à proteção dessa população, com base em evidências georreferenciadas e culturais. 

Referência 11-14: rever. Sugere-se incluir a citação da fonte primária ao invés da citação da fonte secundária. 

Referência 1, 26: rever , os link de acesso não estão funcionando. 

Solicitamos que todos os pontos destacados sejam cuidadosamente atendidos, com especial atenção para: 

Revisão do título e coerência terminológica ao longo do texto (evitar “mortalidade” sem taxas). 

Maior contextualização epidemiológica e sociocultural na introdução. 

Detalhamento metodológico que assegure reprodutibilidade. 

Apresentação mais sintética e visualmente clara dos resultados. 

Discussão crítica, ancorada na literatura, sobre achados e limitações. 

Após a incorporação das modificações, o manuscrito poderá ser reconsiderado para nova rodada de avaliação. 

Recommendation: Major revision

## Second round comments 

Recentemente, você enviou a primeira revisão do manuscrito candidato à publicação na RESS ID 2025-0565.R1 intitulado "Tendência temporal e distribuição espacial dos óbitos por doenças respiratórias infecciosas na população indígena: análise espacial e de séries temporais, Minas Gerais, 2000-2023". Após análise do parecer enviado, verificamos a necessidade de ajustes e complementações. 

Segue abaixo os motivos que justificam essa solicitação: 

Introdução 

Na primeira linha, deve-se alterar o termo “síndromes clínicas”, pois nem toda doença respiratória infecciosa constitui uma síndrome. 

Os autores utilizam como referência para o primeiro parágrafo uma análise sistemática derivada do estudo Global Burden of Disease (GBD). Recomenda-se atualizar os dados sobre a magnitude do problema das doenças respiratórias infecciosas, substituindo a referência inicial e apresentando, no segundo parágrafo, informações derivadas da versão mais recente do GBD. 

Os autores mencionam a relação entre a população indígena e as desigualdades em saúde, porém faltam referências mais explícitas sobre as barreiras estruturais enfrentadas, como: 

dificuldade de acesso geográfico; 

precariedade das unidades básicas de saúde em áreas indígenas; 

carência de profissionais e medicamentos; 

e desassistência em vigilância e diagnóstico. 

Apesar da inclusão de dados sobre densidade domiciliar média, sugere-se complementar com outros indicadores que evidenciem essas desigualdades, conforme o comentário prévio do revisor. 

Uso do termo “mortalidade” 

O comentário anterior permanece parcialmente atendido. Ainda há, na introdução e ao longo do texto, o uso do termo “mortalidade” para descrever o objetivo e a distribuição espacial. 

Considerando que não foram utilizadas estimativas populacionais da população indígena sob risco, o termo pode ser tecnicamente inadequado. Recomenda-se substituir por expressões como “óbitos registrados” ou “número de óbitos”, que descrevem com maior precisão a natureza descritiva dos dados e evitam interpretações equivocadas sobre risco de morte. 

Métodos - Subitem “Variáveis” 

No novo parágrafo adicionado, sugere-se adequar a formatação da letra, mantendo a padronização tipográfica do restante do manuscrito. 

Séries temporais - Imputação de valores 

A estratégia de imputação de valores artificiais (0,10) em anos sem registro de óbitos pode ser metodologicamente questionável, pois tende a distorcer as estimativas de tendência e ampliar variações em séries com baixa frequência de eventos. 

Reitera-se a sugestão de considerar, como alternativa, o uso de média móvel de três pontos, técnica amplamente utilizada para suavizar flutuações aleatórias em séries raras. Caso optem por manter o método atual, sugere-se discutir suas limitações explicitamente na seção de Discussão. 

Discussão 

Os últimos parágrafos ainda não apresentam referências de apoio. Recomenda-se incluir fontes atualizadas que sustentem as interpretações e reforcem a coerência das conclusões apresentadas. 

Agradecemos, mais uma vez, pela sua colaboração e esperamos continuar contando com sua valiosa contribuição nas revisões proferidas. 

Cordialmente, 

Editora Associada da Epidemiologia e Serviços de Saúde: revista do SUS (RESS) 

Recommendation: Minor Revision

